# Growth factor dependent changes in nanoscale architecture of focal adhesions

**DOI:** 10.1038/s41598-021-81898-x

**Published:** 2021-01-27

**Authors:** Karin Legerstee, Tsion E. Abraham, Wiggert A. van Cappellen, Alex L. Nigg, Johan A. Slotman, Adriaan B. Houtsmuller

**Affiliations:** 1 Department of Pathology, Erasmus Medical Center Rotterdam, Rotterdam, 3015 GE the Netherlands; 2 Optical Imaging Centre, Erasmus Medical Center Rotterdam, Rotterdam, 3015 GE the Netherlands

**Keywords:** Cell biology, Cell migration, Cellular imaging

## Abstract

Focal adhesions (FAs) are flat elongated structures that mediate cell migration and link the cytoskeleton to the extracellular matrix. Along the vertical axis FAs were shown to be composed of three layers. We used structured illumination microscopy to examine the longitudinal distribution of four hallmark FA proteins, which we also used as markers for these layers. At the FA ends pointing towards the adherent membrane edge (heads), bottom layer protein paxillin protruded, while at the opposite ends (tails) intermediate layer protein vinculin and top layer proteins zyxin and VASP extended further. At the tail tips, only intermediate layer protein vinculin protruded. Importantly, head and tail compositions were altered during HGF-induced scattering with paxillin heads being shorter and zyxin tails longer. Additionally, FAs at protruding or retracting membrane edges had longer paxillin heads than FAs at static edges. These data suggest that redistribution of FA-proteins with respect to each other along FAs is involved in cell movement.

## Introduction

Cellular adhesion to the extracellular matrix (ECM) is primarily facilitated by focal adhesions (FAs), flat elongated structures 1–2 µm long, 300–500 nm wide and 200–300 nm thick^1,2^. They are macromolecular multiprotein complexes that link the intracellular cytoskeleton to the extracellular matrix. Their linkage to the ECM is mainly mediated by integrins, transmembrane receptors that bind directly to the ECM. Connection of FAs to the cytoskeleton takes place through stress-fibres, a specialised form of F-actin or filamentous actin which is associated with contractile myosin-II. As protein complexes linking two constantly remodelling networks, the ECM and the cytoskeleton, FAs are continuously exposed to force, the strength of which depends on the combination of myosin-II contractility and the stiffness of the ECM. As important regulators of cell adhesion and of force transmission, FAs are crucial to most types of cell migration, including in vitro over a 2D surface. Since migration and cell adhesion are important in many (patho)physiological processes, FAs also play important roles in many such processes, e.g. embryonic development, normal functioning of the immune system and cancer, in particular metastasis^3–5^. FAs are large and diverse macromolecular protein assemblies, with over 200 different reported proteins^4,6^. These include (trans)membrane receptors, adaptor proteins and many different signalling proteins such as kinases, phosphatases and G-protein regulators, which through post-translational modifications add significantly to FA complexity.

It is well established that these diverse proteins are not randomly distributed along the z-axis of the FA but form a layered nanostructure. A seminal study revealed the presence of three different layers: (1), the so-called integrin signalling layer (ISL) closest to the adherent membrane (within ~ 10–20 nm) which includes the cytoplasmic tails of the transmembrane integrin receptors, focal adhesion kinase and paxillin, (2), the actin-regulatory layer (ARL) at the top, where mainly directly actin-binding proteins such as zyxin, vasodilator-stimulated phosphoprotein (VASP) and α-actinin are found, and (3), the force transduction layer (FTL) in between (from ~ 10–20 to ~ 50–60 nm from the adherent membrane) of which talin is the most well-known protein and which is also where vinculin is found^7^. Later studies, using different techniques, confirmed the layered nanostructure of FAs along the z-axis^8–10^.

Here we used the enhanced resolution provided by Structured Illumination Microscopy (SIM) to further examine the distribution of FA proteins within focal adhesions^11^. We selected four hallmark FA proteins which localise to different layers within the previously described layered nanostructure to serve as markers: the large scaffold proteins paxillin (bottom ISL layer) and vinculin (middle FTL layer), and two FA proteins that are closely linked to the actin associated with FAs, zyxin and VASP (top ARL layer). As adaptor proteins, paxillin and vinculin are among the proteins with the most potential binding partners within FAs^6^. In line with their linking, structural, role they are amongst the first proteins to be recruited to assembling FA complexes, especially the directly integrin-binding paxillin^12–16^. Unlike paxillin, vinculin can directly bind actin filaments as well as the actin-binding proteins α-actinin and the ENA/VASP-proteins^17–24^. Zyxin and VASP are recruited to assembling FAs at much later stages than paxillin or vinculin and are more closely linked to actin^15^. In response to mechanical cues and during TGF-β induced EMT zyxin, VASP and vinculin stimulate actin polymerisation in a co-dependent manner^25–31^.

We also studied the effect of increased cellular movement (scattering) on FA nanostructure by stimulating cells with Hepatocyte growth factor (HGF). HGF is known for its involvement in cancer, in particular for its promotion of metastasis through a variety of mechanisms, including strong increases in cell motility^32–37^. Thus, stimulation with HGF strongly increases the motility of cells in both 2D and 3D environments^35,38–40^.

## Results

**Figure 1:**
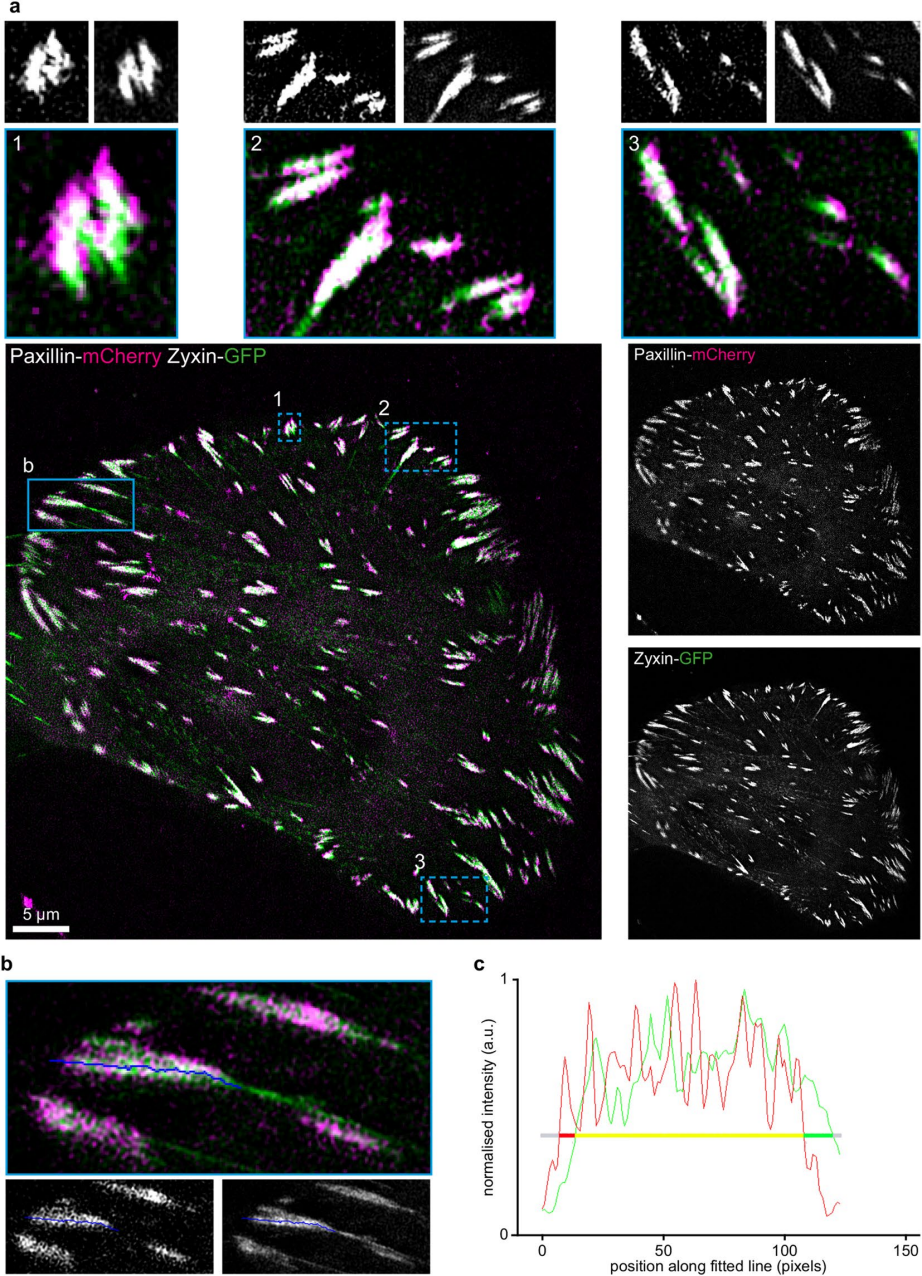
Measuring FA head and tail lengths using structured illumination microscopy. (**a**) Representative SIM image of a U2OS cell co-expressing C-terminally tagged paxillin-mCherry and zyxin-GFP. The bottom row shows the merged image on the left (red channel pseudocoloured magenta) and greyscale images of the indicated separate channels are provided on the right. The three numbered panels in the top row are magnifications of the corresponding blue dashed boxed areas and the greyscale images on top show the red (left) and green (right) channels separately. Contrast is enhanced for visualisation purposes, note that data analysis is always performed on images with original contrast settings. (**b**) Magnification of the indicated blue boxed area in a of the merged (top), red (bottom, left) and green (bottom, right) channels. The blue line was automatically fit to the longitudinal axis of the FA (see Materials and Methods). (**c**) Intensity profiles of the red and the green channels as measured along the line indicated in b, normalised to maximum values. The horizontal line indicates the threshold (40% of maximum intensity, see Materials and Methods). Yellow indicates that both signals are above threshold; red indicates that only the red channel is above threshold; green indicates only the green channel above threshold; grey indicates both channels are below threshold.

**Figure 2:**
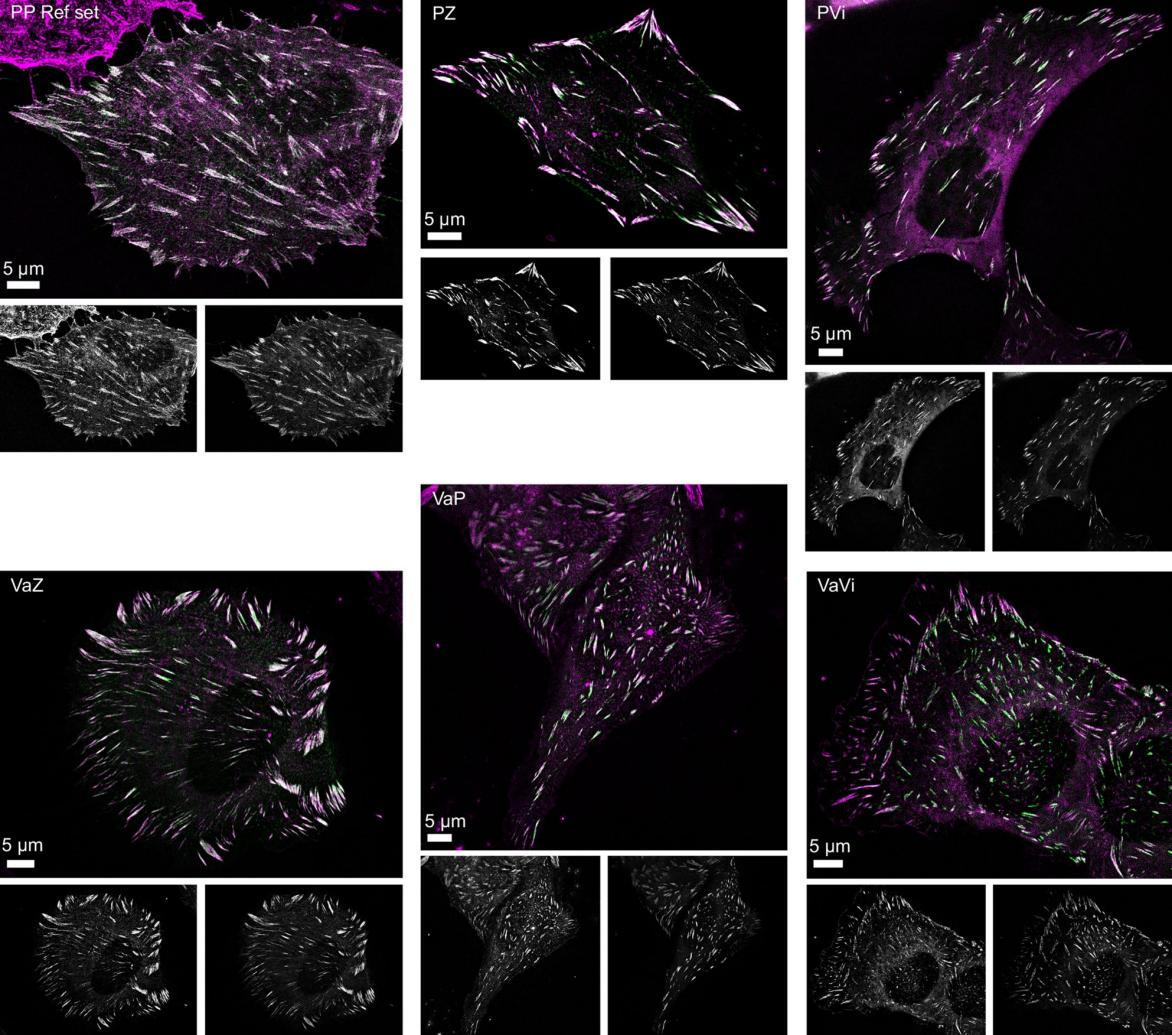
Representative SIM images of U2OS cells co-expressing FA protein pairs. Representative SIM images of U2OS cells co-expressing the indicated protein combinations (P: Paxillin, Vi: Vinculin, Va: VASP, Z: Zyxin), where the first is C-terminally mCherry-tagged and the second is C-terminally GFP-tagged. In the merged image the red channel is pseudocoloured magenta, the grayscale images below each merged image show the red (left) and green (right) channels separately. For visualisation purposes the contrast is enhanced, note that all data analysis is performed on images with unadjusted contrast settings.

The study presented here was motivated by observations we made in pilot experiments using dual colour superresolution (SIM) to visualise the distribution of paxillin-mCherry and zyxin-GFP within focal adhesions. We noted that in many FAs the central part, where paxillin and zyxin colocalised (yellow), was flanked by paxillin (red) at the FA end pointing towards the edge of the adherent membrane, further referred to as ‘head’, and by zyxin (green) at the opposite end, further referred to as ‘tail’ (Fig. 1a). We hypothesised that the observed shift along the long FA axis could be due to the fact that paxillin and zyxin are found in different FA z-layers (see Introduction), which may move with respect to each other during FA function. Therefore, we selected next to paxillin (bottom ISL layer) and zyxin (top ARL layer), two other hallmark FA proteins: VASP which is in the same layer as zyxin, and vinculin which resides in the middle FTL layer. We then used the enhanced resolution of structured illumination microscopy (SIM) to study their distribution within focal adhesions, in both live and chemically fixed cells. Fluorescently tagged constructs of these proteins were previously characterised and shown to be fully functional^7^. They were co-expressed in pairs in human bone cancer (U2OS) cells where they target to FAs, highlighting these in red (mCherry) and green (GFP) fluorescence (Figs. 1a, 2 and supplementary Figs. S1–S6). (note that for overall clarity the red mCherry channel is pseudocoloured magenta in all figures).

**Figure 3:**
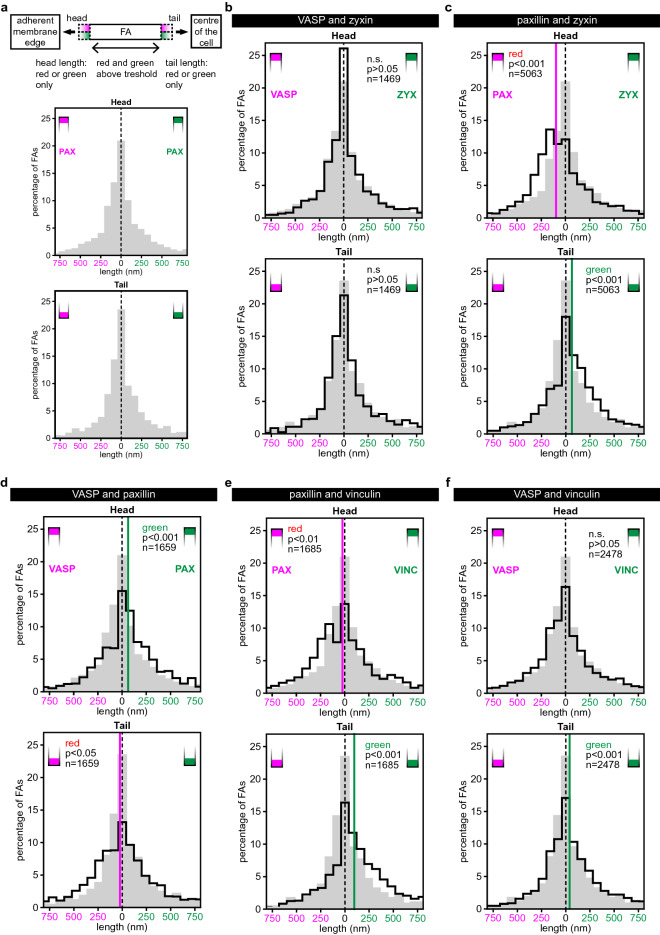
Histograms of the distribution of head and tail protrusion lengths. (**a**) Top panel: schematic representation of an FA in the two colour SIM images clarifying the method. Bottom panels: pax/pax reference histograms showing the distribution of the apparent head (top) and tail (bottom) protrusion lengths measured in the SIM images of the pax/pax reference set of U2OS cells co-expressing paxillin-mCherry and paxillin-GFP. These histograms are further systematically shown in grey in each presented histogram in all figures. (**b**–**f**) Histograms of FA head and tail protrusion lengths for the indicated protein pairs co-expressed in U2OS cells. Red protrusion lengths are plotted from right to left (i.e. negative green protrusion lengths) and green from left to right. The *p* values are generated by comparison of the experimental histogram (black line) and the reference histogram (grey) in two-sided Mann Whitney *U* tests, n represents the number of analysed FAs. Vertical lines: significant median protrusion lengths. Number of imaged cells from b to f: 15, 52, 17, 18 and 23. The pax/pax reference histograms are shown in grey.

To quantitatively study the distribution of pairs of the selected proteins along the long axes of focal adhesions, we developed a semi-automated method (see Materials and Methods). Briefly, in this method a line is fitted along the long axis of FAs and signal intensities for the proteins under investigation are determined along that line (Fig. 1b,c). Next, the head and tail protrusion lengths are determined. To calibrate the method we obtained dual colour SIM images and generated a pax/pax reference set of 1630 FAs in 16 U2OS cells co-expressing paxillin-GFP and paxillin-mCherry (Fig. 2 and Supplementary figure S1). We measured small apparent protrusion lengths both at the heads and at the tails, although mCherry- and GFP-tagged paxillin molecules are expected to be equally distributed within FAs (Fig. 3a and grey distributions in all presented histograms). The observed differences are due to SIM noise and differences in signal-to-noise in the red and green channel, potential chromatic aberrations between the two channels were controlled for by applying channel alignment procedures to all SIM images based on multi-coloured beads (Fig. 1c, Materials and Methods and Supplementary Fig. S7). The pax/pax reference histograms were subsequently generated in the following way: two histograms, one of the distribution of apparent head protrusion lengths and one of apparent tail protrusion lengths were generated: green protrusion lengths were plotted from left to right in the histogram and red protrusion lengths from right to left (‘negative’ green protrusion lengths). These two reference histograms, further referred to as the pax/pax reference histograms, were used to compare histograms generated in the same way from dual colour SIM imaging of the different FA-protein pairs under investigation. If, for example, the red stained protein protrudes at the tail, the histogram of tail protrusions will be shifted to the left with respect to the pax/pax reference histograms. This shift then represents the median tail protrusion length (see Materials and Methods; Fig. 3b–f).

We first performed dual colour imaging SIM experiments on U2OS cells co-expressing the two FA proteins present in the same z-layer (top ARL-layer), zyxin-GFP and VASP-mCherry (note that these and all other constructs were C-terminally tagged) and generated histograms of the distribution of head and tail protrusions. No statistically significant difference was found between these histograms and the reference histograms (Fig. 3b), indicating that zyxin and VASP are similarly distributed along the FA. We then imaged each of these in combination with paxillin (bottom ISL-layer), expecting similar results in both combinations. Paxillin-mCherry and zyxin-GFP showed significantly shifted histograms indicating the presence of paxillin head protrusions and zyxin tail protrusions (Fig. 3c). As expected, similar results were obtained for paxillin-GFP and VASP-mCherry, *i.e.* paxillin head protrusions and VASP tail protrusions (Fig. 3d). Note that the use of paxillin-GFP in one, and paxillin-mCherry in the other experiment (colour swap), demonstrated that protrusion measurements are not influenced by differences in imaging red or green dyes, nor by differences in the photo-physical properties of these dyes. Paxillin-mCherry and Vinculin-GFP also showed significantly shifted histograms indicating paxillin head protrusions and vinculin tail protrusions (Fig. 3e). When VASP-mCherry and vinculin-GFP were co-expressed, no significant head protrusions were observed (*i.e.* the histogram was not statistically different from the reference histogram), but at the tail, vinculin protruded significantly further than VASP (Fig. 3f). In summary, at FA heads paxillin protruded significantly further than vinculin, zyxin or VASP, while these latter three protruded significantly further at the tails with vinculin protruding the furthest.

**Figure 4:**
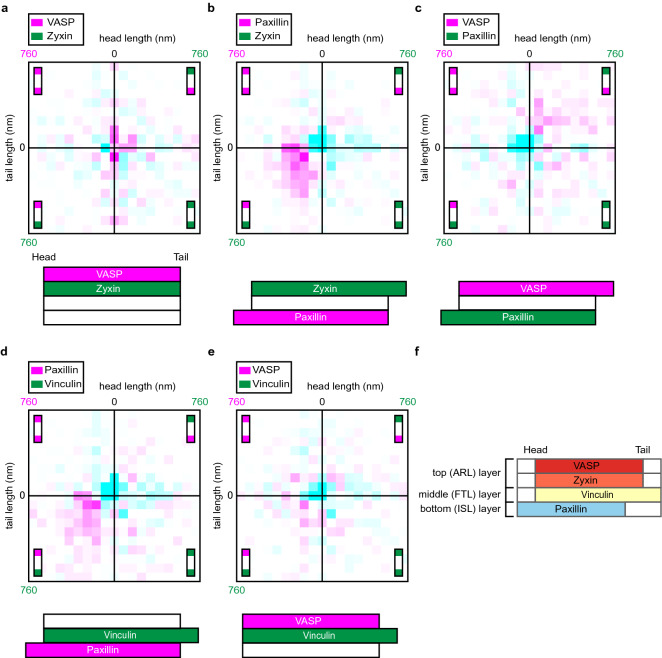
2D-histograms combining the head and tail protrusion lengths of individual FAs. (**a**–**e**) The data presented in Fig. 3 shown as 2D-histograms combining both the head (horizontal axis) and the tail (vertical axis) protrusion lengths for each individual FA. Colours represent the difference between the relative distributions of the reference set and the indicated protein pair. White: below threshold (see Materials and Methods), magenta: increase relative to reference set, cyan: decrease relative to reference set. Colour intensity represents magnitude of the change (for scale see Materials and Methods). Cartoons below provide a schematic summary of the data (not to scale). (**f**) A side view model for the longitudinal distribution of proteins along FAs based on the data from the different co-expressed FA protein pairs and the z-layer each protein is found in according to the seminal paper by Kanchanawong et al.^7^.

We subsequently created 2-D histograms of the relative frequencies of combined head/tail lengths of individual FAs compared to the pax/pax reference histograms (Fig. 4, Supplementary Fig. S8). These provide a visualisation of the relative frequency of the combination of a specific head and tail length compared to the relative frequency at which that combination occurred in the pax/pax reference histograms. Values higher than in the pax/pax reference histograms are shown in magenta and lower values in cyan. Colour intensity indicates the magnitude of the differences (see Materials and Methods for scale details). The VASP/zyxin protein set showed a distribution similar to the pax/pax reference histograms, again indicating neither protein protruded significantly at either FA end (Fig. 4a). In the data sets including paxillin (Fig. 4b–d), the relative frequency of FAs with the combination of none to very short head and tail lengths was decreased compared to the pax/pax reference histograms (cyan clusters at the centres of the histograms). The frequency of FAs with paxillin heads in combination with zyxin, vinculin or VASP tails was increased (magenta clusters in the respective quadrants of the histograms). The 2D-histogram of the VASP/vinculin set (Fig. 4e) clearly shows that the frequency of FAs with short head and tail protrusions is reduced (cyan cluster at the centre of the histogram), but the observed increases are small (no dark magenta) and they do not seem to cluster anywhere in the histogram. This is to be expected because neither protein extended significantly at the FA head, and only at the tail vinculin protruded significantly (Fig. 3f). Because there is no preference for either protein at the head, the increased frequency of FAs with vinculin tails is distributed over a large part of the lower half of the histogram, not clustered in a particular quadrant as is the case for the protein sets with paxillin, making the differences at individual bins less pronounced.

### FA heads shorten and tails lengthen during scattering

**Figure 5:**
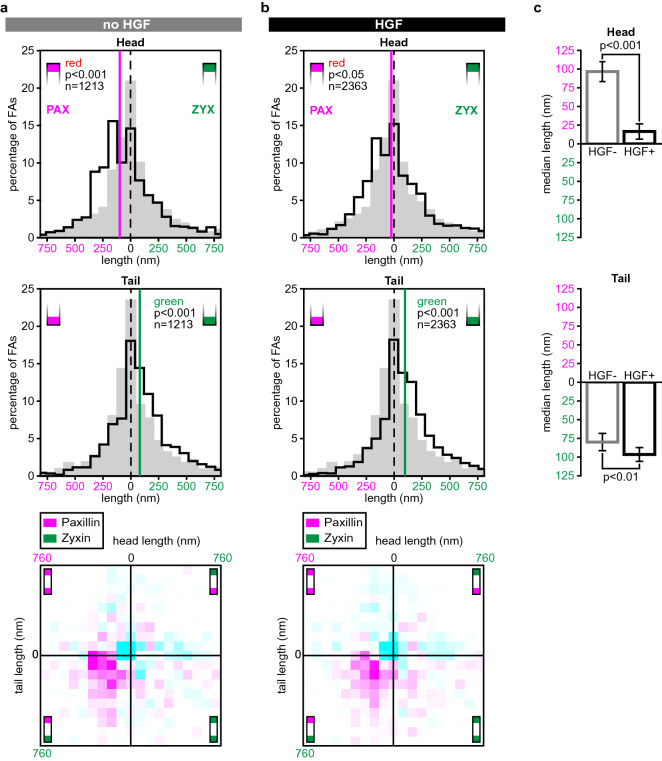
HGF treatment alters the longitudinal distributions of paxillin and zyxin along FAs. (**a**) Histograms of head (top) and tail (middle) protrusion lengths for 12 U2OS cells co-expressing paxillin-mCherry and zyxin-GFP and subjected to overnight serum-starvation. Reference histograms showing measured protrusion lengths in the pax/pax reference set are shown in grey. *p* values generated compared to the reference set (two-sided Mann Whitney *U* tests), n gives number of analysed FAs. Vertical lines: medians. Bottom: 2D-histogram combining head and tail protrusion lengths for each individual FA. Colours represent the difference compared to the relative distributions of the reference set. White: below threshold, magenta: increase relative to reference set, cyan: decrease relative to reference set. Colour intensity represents magnitude of the difference (for scale and threshold see Materials and Methods). (**b**) Histograms of head (top) and tail (middle) protrusion lengths for 21 U2OS cells co-expressing paxillin-mCherry and zyxin-GFP, subjected to overnight serum-starvation and treated with HGF at least three hours prior to imaging. Grey histograms, vertical lines, n-numbers and statistical tests as above. Bottom: 2D-histogram combining head and tail protrusion length for each individual FA. Colours as above. (**c**) Comparison of head (top) and tail (bottom) protrusion lengths for the serum starved HGF minus (grey outline) and the serum starved and HGF treated (black outline) cells, *p* values are generated by two-sided Mann Whitney *U* tests. Errorbars show standard error of the mean (SEM).

Next, we examined the effect of HGF-induced scattering on the distribution of paxillin and zyxin along FAs. HGF, also known as the scattering factor, induces a scattering response in epithelial cells, including in U2OS cells^35,38–40^. During scattering cells display increased motility and undirected migration. In serum starved cells, not incubated in the presence of HGF (Fig. 5a) the same patterns were seen as before (Figs. 3c, 4b), with paxillin protruding significantly more often at the head and zyxin at the tail. Following HGF-stimulation however, the paxillin enriched head became significantly shorter (*p* < 0.001) to the point of nearly disappearing, with a length that is only just significantly different from the pax/pax reference histograms (*p* = 0.045, Fig. 5b,c). At the same time, following HGF-treatment the length of the zyxin tail region was significantly increased (*p* < 0.01) (Fig. 5b,c). In the unstimulated cells the strongest increases in the 2-D histograms were seen further to the left than in the HGF-treated cells, indicating longer paxillin heads in combination with zyxin tails. Thus the differential longitudinal distribution of FA proteins within FAs is altered in response to HGF, with paxillin heads shortening and zyxin tails lengthening.

### FA head lengths correlate with membrane motility

**Figure 6:**
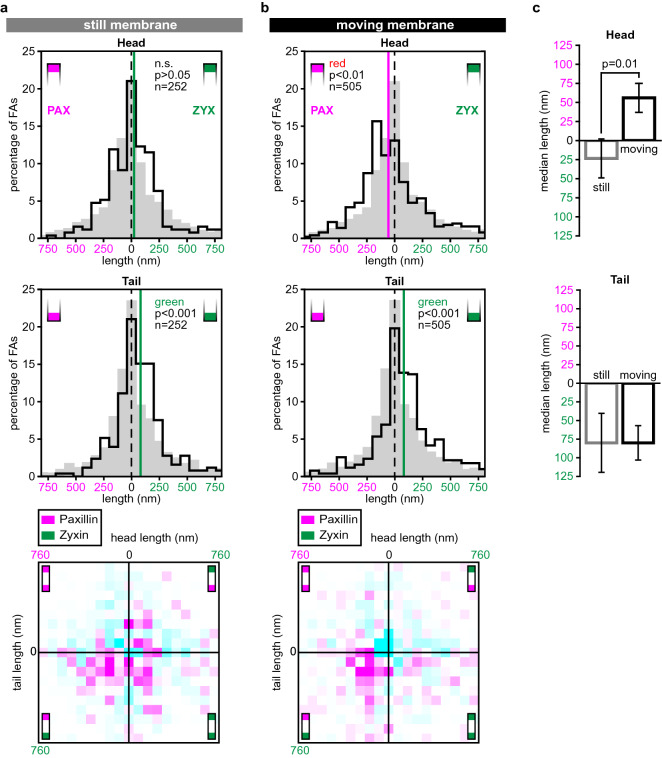
The longitudinal distributions of paxillin and zyxin differ for FAs near static compared to moving membrane edges. (**a**) Histograms of head (top) and tail (middle) protrusion lengths for FAs near static membrane edges (not protruding or retracting) in U2OS cells co-expressing paxillin-mCherry and zyxin-GFP subjected to overnight serum-starvation and HGF-treatment. Pax/pax reference histograms showing the distribution of the pax/pax reference set are plotted in grey. *p* values generated compared to the reference histograms (two-sided Mann Whitney *U* tests), n gives number of analysed FAs. Vertical lines: median protrusion lengths. Bottom: 2D-histogram combining head and tail protrusion lengths for each individual FA. Colours represent the difference compared to the relative distributions of the reference set. White: below threshold, magenta: increase relative to reference set, cyan: decrease relative to reference set. Colour intensity represents magnitude of the difference (for scale and threshold see Materials and methods). (**b**) Histograms of head (top) and tail (middle) protrusion lengths for FAs near moving membrane edges. Grey histograms, vertical lines, n-numbers and statistical tests as above. Bottom: 2D-histogram combining head and tail protrusion length for each individual FA. Colours as above. (**c**) Comparison of head/tail length for FAs near still (grey) and moving (black) ventral membrane edges, *p* values are generated by two-sided Mann Whitney *U* tests. Errorbars show standard error of the mean (SEM).

Finally, we examined whether the lateral distribution of paxillin and zyxin along FAs correlated with the motility of the nearest ventral (adherent) membrane edge. Nine HGF-stimulated cells were reimaged approximately 30 min after the measurements shown in Fig. 5b, to analyse the movement of their ventral membrane edges (see Materials and Methods). 505 FAs were located close to a moving membrane edge and 252 FAs were located near a static membrane edge, while 266 FAs were disregarded from this analysis because of a more inward cellular position. At FAs near static membrane edges the paxillin enriched head was undetectable, which differed significantly from FAs near protruding or retracting edges that retained their paxillin enriched heads (Fig. 6). In contrast, FAs near both moving and still membrane edges have similar zyxin enriched tails. In the 2-D histograms this translates into the increases being spread all over the lower half of the histogram for static membrane edges. However, for FAs near moving membrane edges the strongest increases are clearly clustered in the lower left quadrant indicating FAs with paxillin heads combined with zyxin tails are seen more frequently.

## Discussion

**Figure 7:**
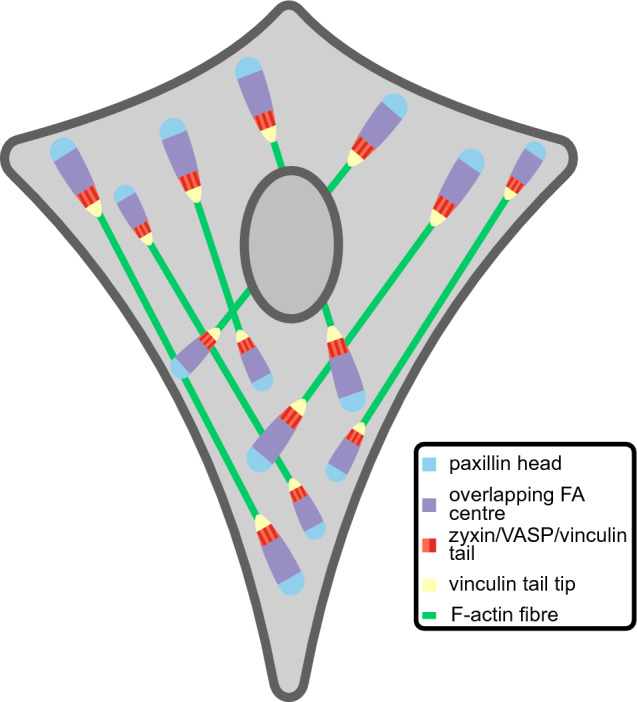
Schematic model summarizing the data. Schematic representation of the distribution of hallmark FA proteins paxillin, vinculin, zyxin and VASP along the long axis of FAs within cells. For clarity the model is not drawn to scale.

Here we examined large numbers of FAs in cells co-expressing hallmark FA proteins in various combinations and showed that paxillin frequently protrudes at FA heads, zyxin and VASP at tails and vinculin at tail tips (Figs. 3, 4, 7). Since these proteins each are present in a specific z-layer our data suggests these layers are shifted relative to each other, where in the most frequently observed configuration the layer closest to the ventral membrane (ISL) extends furthest at FA heads, the middle layer (FTL) at the tail tips and the top layer (ARL) at the tail (Fig. 4f).

We used multi-colour beads to perform separate channel alignments for all images (Materials and Methods and Supplementary Fig. S7). To rule out that other differences between the two dyes such as their different signal-to noise ratios impact their measured distributions, we performed a number of controls. As a first control, we created pax/pax reference histograms by imaging cells co-expressing red- and green-tagged versions of a single FA protein, paxillin (Fig. 3a and grey distributions in all presented histograms). This allowed statistical testing of whether the head/tail lengths we observe for the image data sets of U2OS cells co-expressing different proteins are significantly different from those in the pax/pax reference histograms. Secondly, when we examined the longitudinal distribution of two proteins from the same z-layer, zyxin and VASP, we found histograms that were not significantly different from the pax/pax reference histograms, showing that VASP and zyxin are similarly distributed along FAs (Figs. 3b, 4a). We used this finding to perform an internal colour swap control experiment where we swapped the tag of paxillin when comparing with zyxin and VASP respectively (paxillin-mCherry/zyxin-GFP and VASP-mCherry/paxillin-GFP) (Fig. 3c vs 3d and 4b vs 4c). We refer to these two measurements as ‘internal control experiment’ because they not only serve as controls but also provide experimental data. Since VASP and zyxin have the same longitudinal distribution (Fig. 3b) this should produce similar head and tail lengths but in opposite colours, which is indeed what we observed (Figs. 3c,d, 4b,c).

Our observation of vinculin protruding at tail tips corresponds remarkably well to previous studies showing that talin is stretched at these tips^7^, since stretching of talin has been shown to unmask cryptic vinculin binding sites^41^. In other studies non-homogeneous protein distributions were also shown along FAs. For example, studies on large FAs at the trailing end of actively migrating cells showed that binding dynamics of paxillin vary along them^42–44^. For vinculin, it has been reported that its active form is enriched at the proximal FA tip (the tail) and its inactive form at the opposite FA end (the head)^45,46^. Also, active and inactive integrins were recently shown to form discrete nanoclusters^47^. Recently, we showed using FRAP and photoswitching in confocal mode (not for single molecule microscopy) that long term immobile paxillin and vinculin form small clusters within the FA complex that are enriched at the proximal half of the FA^48^. Lastly, in specialised FAs unique to pluripotent stem cells, termed cornerstone FAs, β5-integrins and talin were shown to be specifically enriched around the edges of these FAs, while a third protein, the adaptor protein kank2, was shown to accumulate near these FAs specifically at the distal end^10^.

We were also able to show that head and tail compositions were altered during HGF-induced scattering. HGF, or the scatter factor, is the natural ligand for the tyrosine receptor encoded for by the proto-oncogene c-Met^49–51^. The U2OS cells we used express the Met receptor^33^ and stimulation with HGF has been shown to strongly increase the motility of U2OS cells in both 2D and 3D environments^35,38–40^. During HGF-induced scattering we observed that paxillin heads were shorter and zyxin tails longer than in unstimulated cells. Additionally, for the HGF-stimulated cells, FAs at protruding or retracting membrane edges had longer paxillin heads than FAs at static edges. Taken together these findings either suggest that the investigated proteins redistribute within their layers or that the layers shift with respect to each other as a whole, and that this plays a role in both overall cell movement and membrane movement.

With regard to the mechanism underlying the protein (re)arrangements along FAs, it is tempting to speculate involvement of actin (Supplementary Fig. S9) for two reasons. Firstly, paxillin is the only protein investigated here that does not directly bind actin and also the only protein absent from the tail region. Secondly, the tail protrudes at the FA tip where actin stress fibres enter FAs^44,52–54^ (Supplementary Fig. S9). The absence of paxillin at the FA tail suggests that the FA tail is more loosely attached to the extracellular matrix, and then instead may form an easily accessible handle for actin to hold the FA. It may also be that over time paxillin is slightly pulled apart longitudinally from the other three proteins by the force exerted on those by the actin stress fibre, as all three are linked to actin. In the bottom layer, paxillin links the focal adhesion to the ECM-bound integrins and stays in place, in that way also explaining the protrusion of paxillin at the head. Alternatively, the presence of the actin stress fibre at FA tails could be facilitating the binding of proteins directly binding actin (located in the top two layers), by providing a second binding platform for these proteins next to the FA complex itself.

## Materials and methods

### Cell culture

U2OS cells were kindly provided by Dr. M.W. Paul and previously used for instance in Sanchez et al^55^ and Legerstee et al^48^. Cells were cultured in a humidified environment at 37 °C and 5% CO_2_ in phenol-red free DMEM (Lonza), supplemented with 10% FCS (Gibco), 2 mM L-glutamine (Lonza), 100 U/ml Penicillin, 100 µg/ml Streptomycine (Lonza) and, when maintaining stable cell lines, with 100 mg/ml G418. Transfections were performed using Fugene (Promega) followed by selection with G418 when creating stable cell lines. Stable cell lines were co-transfected with a second fluorescently labelled construct. For experiments 24 mm round glass coverslips were coated overnight at 4 °C with PureCol bovine collagen type I (Advanced BioMatrix) at a final concentration of 10 µg/ml. For channel alignment purposes TetraSpeck Microspheres 0.1 μm (Thermo Fisher Scientific) were added to the coating. Cells were seeded onto the coated coverslips 16–24 h after transfection, then 24–48 h later imaged or chemically fixed (4% Paraformaldehyde), mounted using VECTASHIELD Mounting Medium (Vector Laboratories) and stored at 4 °C. For experiments examining the effects of HGF-induced scattering, prior to imaging cells were exposed to overnight serum starvation in 0.5% FCS containing DMEM, which 3–6 h prior to imaging was replaced with DMEM with 0.5% FCS (control) or DMEM with 0.5% FCS and 25 ng/ml HGF (HGF-treated).

### Mammalian expression vectors

All fluorescent protein expression vectors were constructed using N1 (Clontech-style) cloning vectors. They are based on mTurquoise or eGFP-tagged fusion constructs that have been previously characterised biochemically and/or cell biologically in the literature.

The Zyxin-GFP, Vinculin-GFP and Paxillin-GFP vectors were previously described^48^. The VASP-mCherry and Paxillin-mCherry constructs were based on the VASP-mTurquoise (Addgene 55585) and Paxillin-mTurquoise (Addgene 55573) vectors, respectively. These use the multiple cloning site as a linker region between the protein and mTurquoise, hampering a simple colour swap. To still allow sticky-end ligation the protein, fluorescent label and empty vector backbone were all separately isolated as restriction fragments. These were ligated using sticky ends in 2 steps: (1) the protein to the fluorescent label and (2) the created insert into the vector backbone. This strategy was applied in the following manner:

For VASP-mCherry: The vector backbone was isolated from Paxillin-mTurquoise as an AgeI NotI fragment, VASP from VASP-mTurquoise as an AgeI BamHI fragment and mCherry from pmCherry N1 (Clontech) as a BamHI NotI fragment.

For Paxillin-mCherry: The vector backbone was isolated from Paxillin-mTurquoise as a BamHI NotI fragment, paxillin from Paxillin-mTurquoise as a BamHI HindIII fragment and mCherry from pmCherry as a HindIII NotI fragment.

All constructs were checked through sequencing, one silent mutation was found (Paxillin-GFP/Paxillin-mCherry bp1461 C to G). Furthermore, we confirmed all constructs localize properly (Fig. 2 and Supplementary Fig. S1–S6).

### Imaging

All data was acquired on a Zeiss Elyra PS1 system, an integrated microscope system with a structured Illumination unit and a LSM 780 confocal unit using a 63 × 1.4NA oil objective. For live-cell imaging, cells were maintained at 37 °C and 5% CO2 using a stage-top incubator (Tokai Hit). For 3D-SIM imaging a grating was present in the light path. The grating was modulated in 5 phases and 5 rotations, and multiple z-slices were recorded with an interval of 110 nm on an Andor iXon DU 885, 1002 × 1004 EMCCD camera. GFP and mCherry were excited using 488 nm and 561 nm 100 mW diode lasers together with a BP 495–575 or 570–650 nm filter and a LP 750 nm filter. Raw images were reconstructed using the Zeiss Zen 2012 software. To correct for chromatic aberration TetraSpeck beads were embedded within the collagen-coating of the coverslips. Each TetraSpeck bead is loaded with four fluorescent dyes of different colours. These beads were used to perform channel alignment in x, y and z on each image separately using the channel alignment tool from the Zeiss Zen 2012 software. Cells were selected for imaging only if at least one TetraSpeck bead was present within the field of view. For the experiments looking at the effects of membrane motility, time lapse data was obtained for all positions using the LSM 780 confocal unit on the same microscope system at 10–30 min intervals prior to the SIM imaging. Additionally either all positions were imaged at 15–30 min intervals in confocal mode during the SIM imaging or the cells were reimaged in SIM mode 30 min after their original SIM imaging.

### Data analysis

#### Image analysis

Data was analysed in the ImageJ software package within the Fiji framework^56^, using a series of macros we developed for this. From the reconstructed and channel aligned SIM data average projections were made of all z-slices (typically 3) with clearly visible FAs. A macro was used to roughly select the outlines of the FAs. Briefly, from a duplicate of the data to which a Gaussian blur with low σ was applied, another duplicate of the data to which a Gaussian blur with higher σ was applied, was subtracted. FAs were selected based on the Fiji build-in ‘Otsu-dark’ threshold, outlines were individually assessed and where needed corrected (mainly separation of touching FAs). Once all FAs were outlined a second macro was used to fit a line through the longitudinal axis of each FA. The ‘fit ellipse’ option was applied to find the centre of the FA, and the angle between the horizontal axis of the image and the longest axis of the FAs. These were used to fit a line through the longitudinal axis of each FA by fitting a Gaussian to the intensity profile measured in a line perpendicular to the longest axis of the FA in the green channel. The fitted lines were terminated at both ends once the mean intensity of a 3 by 3 pixel sized box in both the green and the red channels fell below 30% of their maximum intensity along the line. For all FAs, intensity profiles along the fitted lines for both channels (red and green) were obtained. Distal and proximal ends were distinguished by determining which end was closest to a rectangle drawn around the cell (i.e. the distal FA end which we refer to as ‘the head’ in the manuscript).

#### Measuring FA head and tail protrusion lengths

The intensity profiles were loaded into R using the RStudio (R development Core Team, 2016). Values were marked as positive for the channels based on a variable threshold (40% of the maximum value of the respective channel after application of a median filter with a window size of 5 pixels). The longest stretch of pixels along the line positive for both channels was determined, i.e. the main stretch of FA where both proteins overlap. This was hampered by the way noise manifests in reconstructed SIM images, as coloured noise due to the Fourier transformations applied in the SIM reconstruction, where even within bright high intensity patches small clusters of low intensity pixels are seen. To compensate for this, a gap of maximally 4 pixels (160 nm) negative for either/both channel(s) was allowed within the main stretch. The intensity data of the pixels surrounding the main stretch was examined to determine the FA head and tail lengths (see Figs. 1c, 3a). If the pixel bordering the main stretch was positive for one of the channels, the identity of this channel and the number of consecutive pixels positive for this channel (again allowing for a gap of maximally 4 pixels) were taken as the head or tail lengths. Note that for conversion of these lengths to nanometres the position of the pixels was also taken into account, so the distance between 2 pixels in a straight line is 40 nm, but between two diagonal pixels is the square root of 3200, or ~ 57 nm. If the pixel bordering the main stretch was negative for both channels, the corresponding head or tail protrusion length was zero.

#### Reference set of paxillin-GFP and paxillin-mCherry

For analysis of the experimental data we first constructed a pax/pax reference set by imaging and analysing 1630 FAs in 16 U2OS cells co-expressing paxillin-mCherry and paxillin-GFP which fully overlap. However, as expected, due to noise and other possible inaccuracies in the images, we still measured apparent head and tail lengths. We found a small bias of 57 nm of measuring green head/tail lengths, for which we corrected all further measurements.

#### Representation and statistical analysis of the experimental data

The experimental data of all investigated protein pairs were plotted together with the data from the pax/pax reference set as histograms with a bin size of 80 nm, with the measured head or tail red lengths to the left of zero and the measured head or tail green lengths to the right. This allowed us to test whether proteins stuck out significantly at the head or tail in comparison to the pax/pax reference set by using two-tailed Mann–Whitney *U* test.

We also generated 2-D histograms to plot the head and tail lengths of the same FA as a single 2-D data point. 2-D histograms had 80 by 80 nm bins. The 2-D distribution of the pax/pax reference set in the 2-D histogram was subtracted from the distribution of the protein set of interest and the distribution of the resulting increases and decreases (in percentage point) relative to the pax/pax reference set were visualised as a 2-D histogram using a colour look up table (LUT), where values between 0 and 123 (corresponding to decreases of 3.45 to 0.13 percentage point) form a continuous range from cyan to white, values between 133 and 255 (corresponding to increases of 0.14to 3.44 percentage point) a continues range from white to magenta and the intermediate values of 124 to 132 (corresponding to increases/decreases of less than 0.11 percentage point) are kept white for clarity.

## Supplementary Information

Supplementary Figures.
