# Supplementary material for: Growth factor dependent changes in nanoscale architecture of focal adhesions
Source: Sci Rep. 2021 Jan 27;11:2315. doi: 10.1038/s41598-021-81898-x (PMC7841166; doi:10.1038/s41598-021-81898-x)
Supplement: Supplementary file 1 — Supplementary Figures. [file 41598_2021_81898_MOESM1_ESM.pdf]

Growth factor dependent changes in nanoscale architecture of focal adhesions

Karin Legerstee[1], Tsion E. Abraham[2], Wiggert A. van Cappellen[2], Alex Nigg[1], Johan A. Slotman[2], Adriaan B. Houtsmuller*[1,2]

1 Department of Pathology, Erasmus Medical Center Rotterdam, Rotterdam, 3015 GE, the Netherlands

2 Optical Imaging Centre, Erasmus Medical Center Rotterdam, Rotterdam, 3015 GE, the Netherlands

* Correspondence: a.houtsmuller@erasmusmc.nl

S1

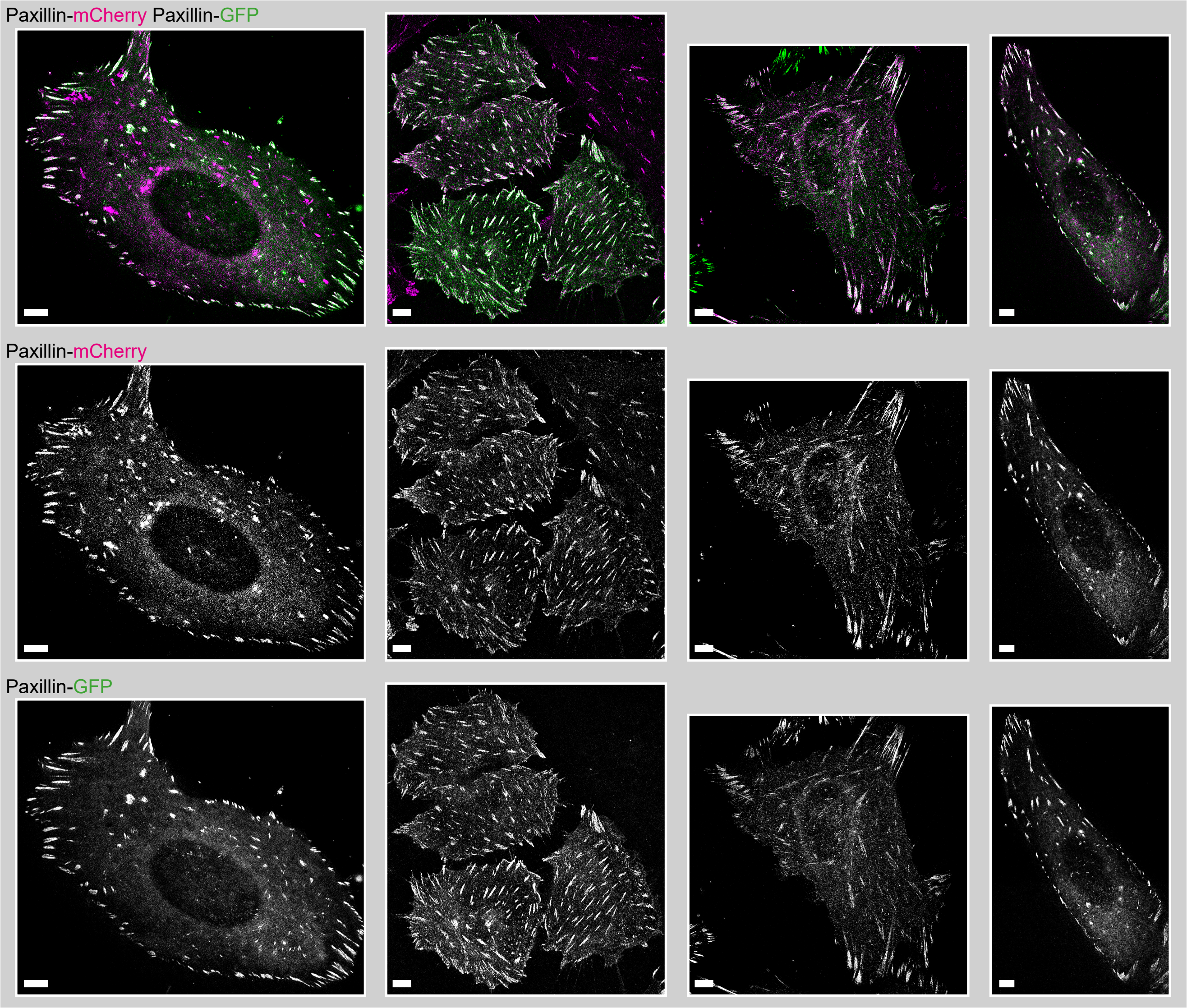

Fig. S1 Representative SIM images of U2OS cells coexpressing Paxillin-mCherry and Paxillin-GFP.
Merged (top), red (middle) and green (bottom) channels, red channel is pseudocoloured magenta in merge images. Scale bar 5 µm. For visualisation purposes the contrast of the channels is enhanced (note that image analysis is performed on images with unaltered contrast settings).

S2

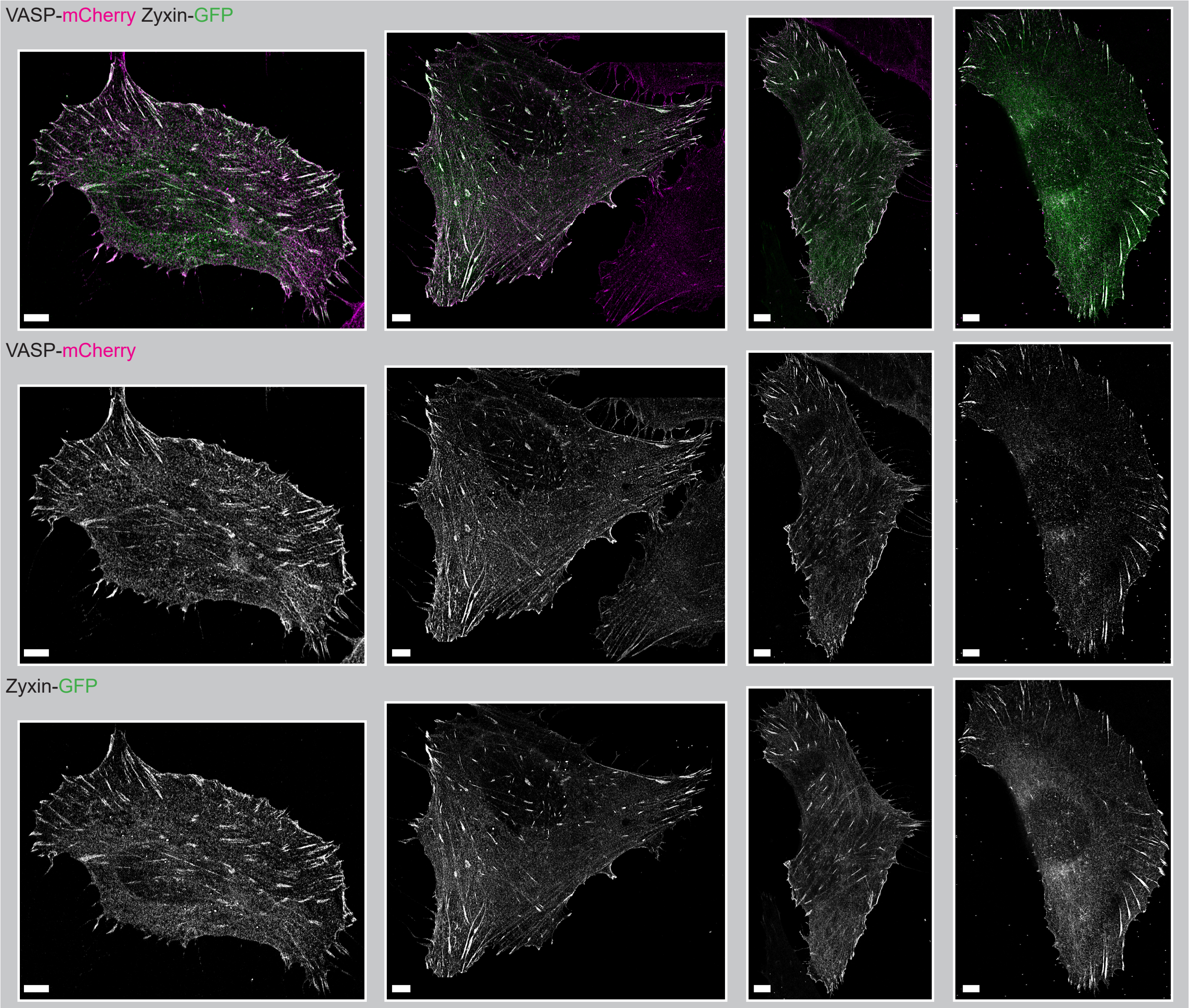

Fig. S2 Representative SIM images of U2OS cells coexpressing VASP-mCherry and Zyxin-GFP.
Merged (top), red (middle) and green (bottom) channels, red channel is pseudocoloured magenta in merge images. Scale bar 5 µm. For visualisation purposes the contrast of the channels is enhanced (note that image analysis is performed on images with unaltered contrast settings).

S3

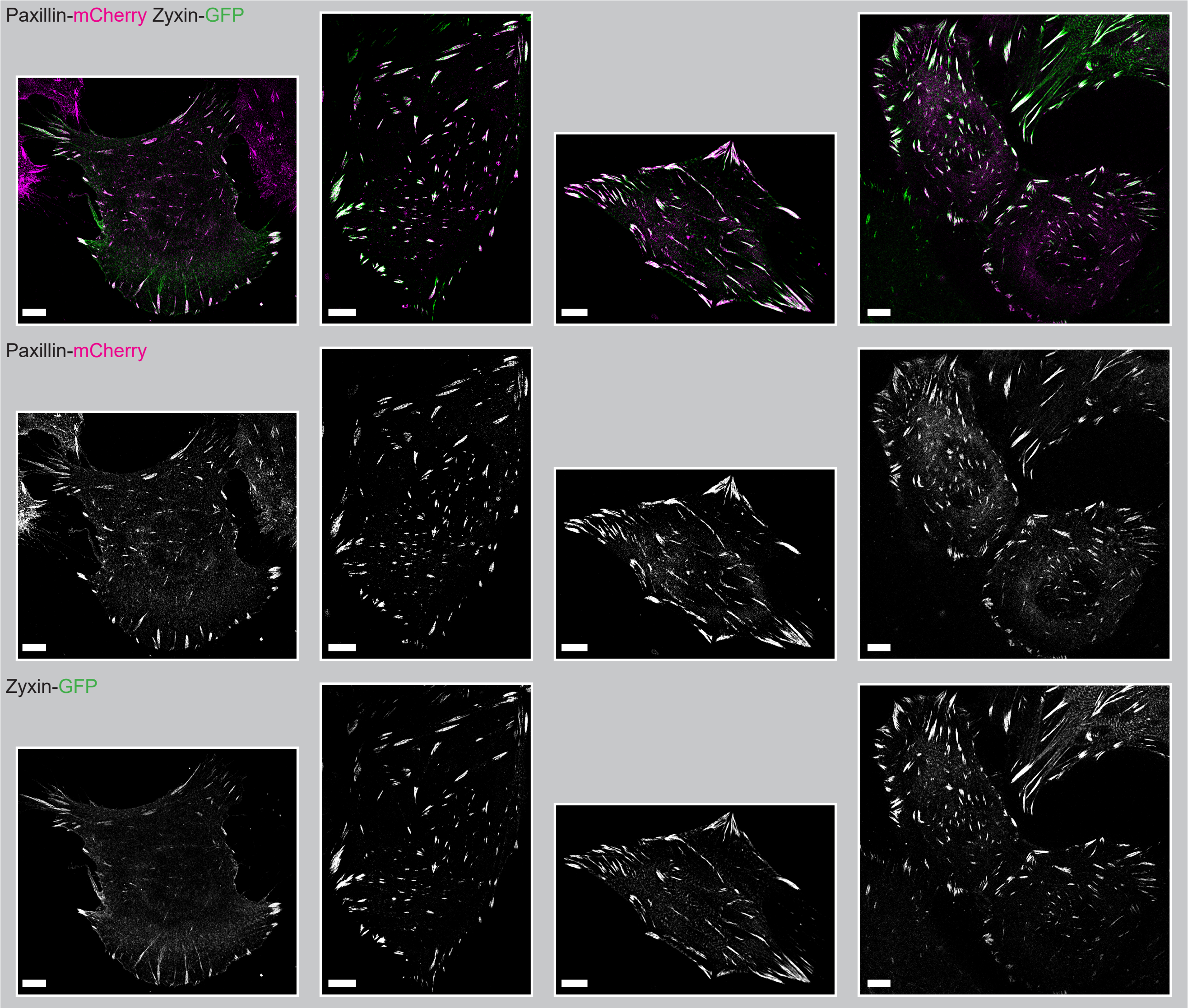

Fig. S3 Representative SIM images of U2OS cells coexpressing Paxillin-mCherry and Zyxin-GFP.
Merged (top), red (middle) and green (bottom) channels, red channel is pseudocoloured magenta in merge images. Scale bar 5 µm. For visualisation purposes the contrast of the channels is enhanced (note that image analysis is performed on images with unaltered contrast settings).

S4

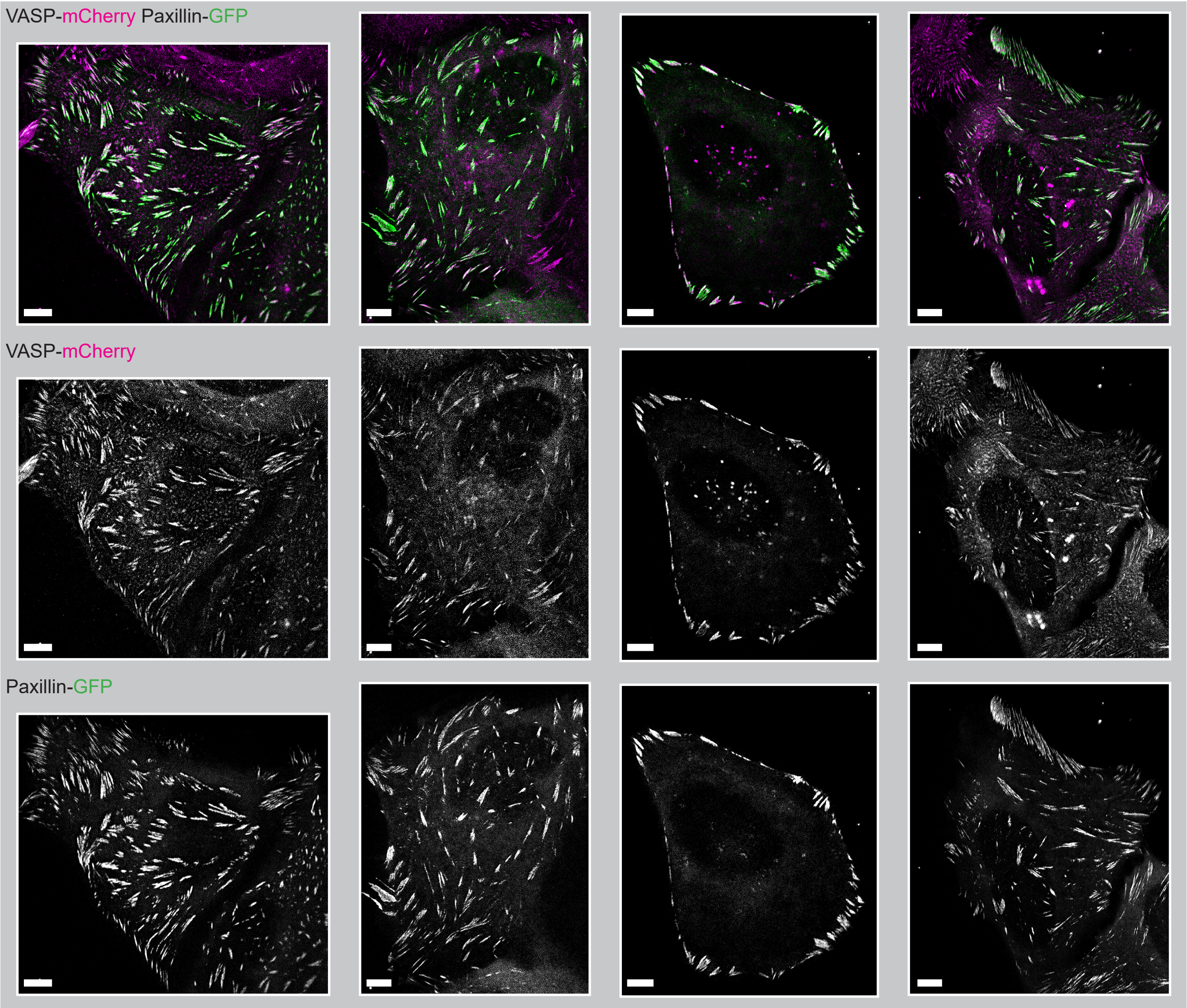

Fig. S4 Representative SIM images of U2OS cells coexpressing VASP-mCherry and Paxillin-GFP. Merged (top), red (middle) and green (bottom) channels, red channel is pseudocoloured magenta in merge images. Scale bar 5 µm. For visualisation purposes the contrast of the channels is enhanced (note that image analysis is performed on images with unaltered contrast settings).

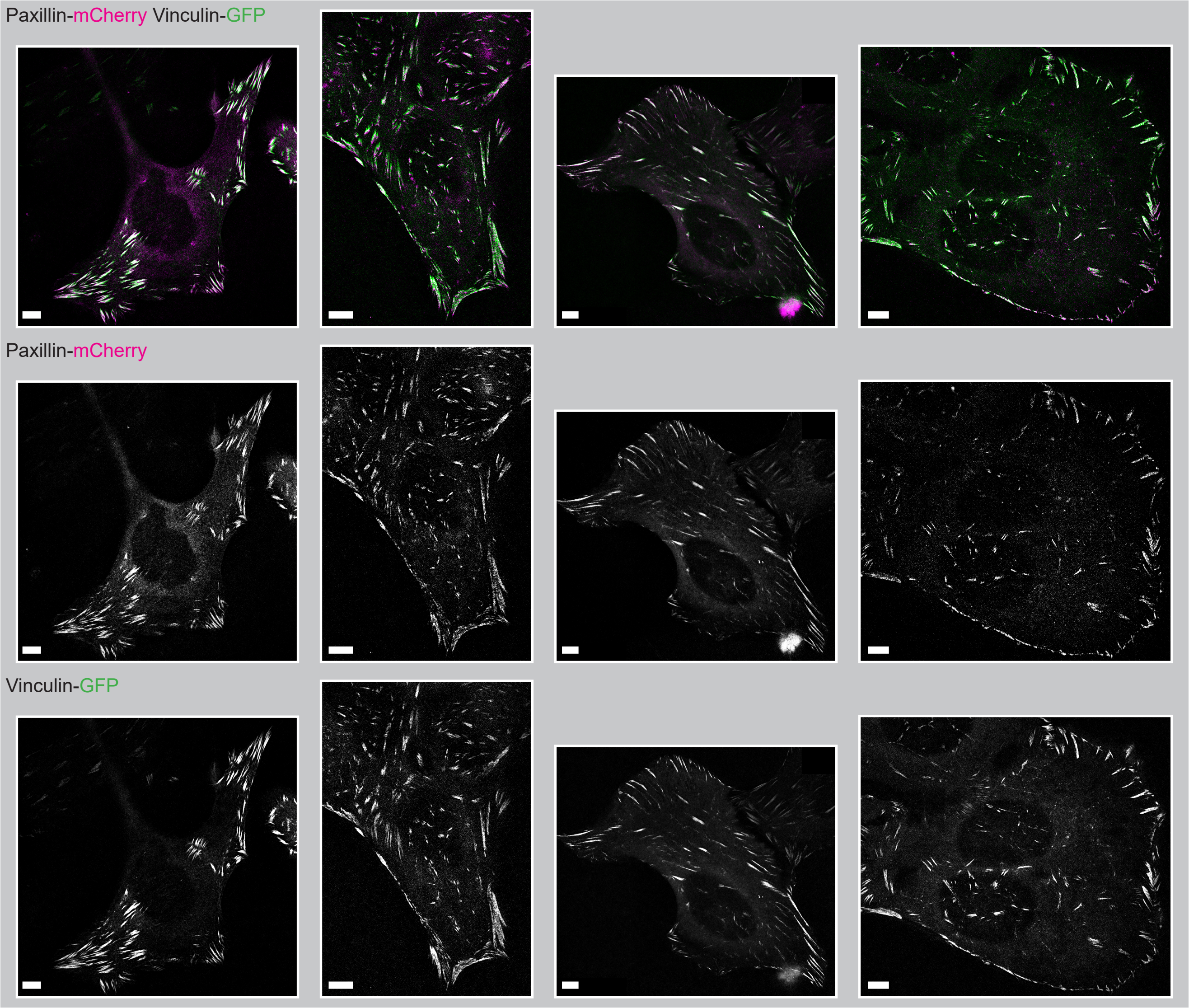

Fig. S5 Representative SIM images of U2OS cells coexpressing Paxillin-mCherry and Vinculin-GFP. Merged (top), red (middle) and green (bottom) channels, red channel is pseudocoloured magenta in merge images. Scale bar 5 µm. For visualisation purposes the contrast of the channels is enhanced (note that image analysis is performed on images with unaltered contrast settings).

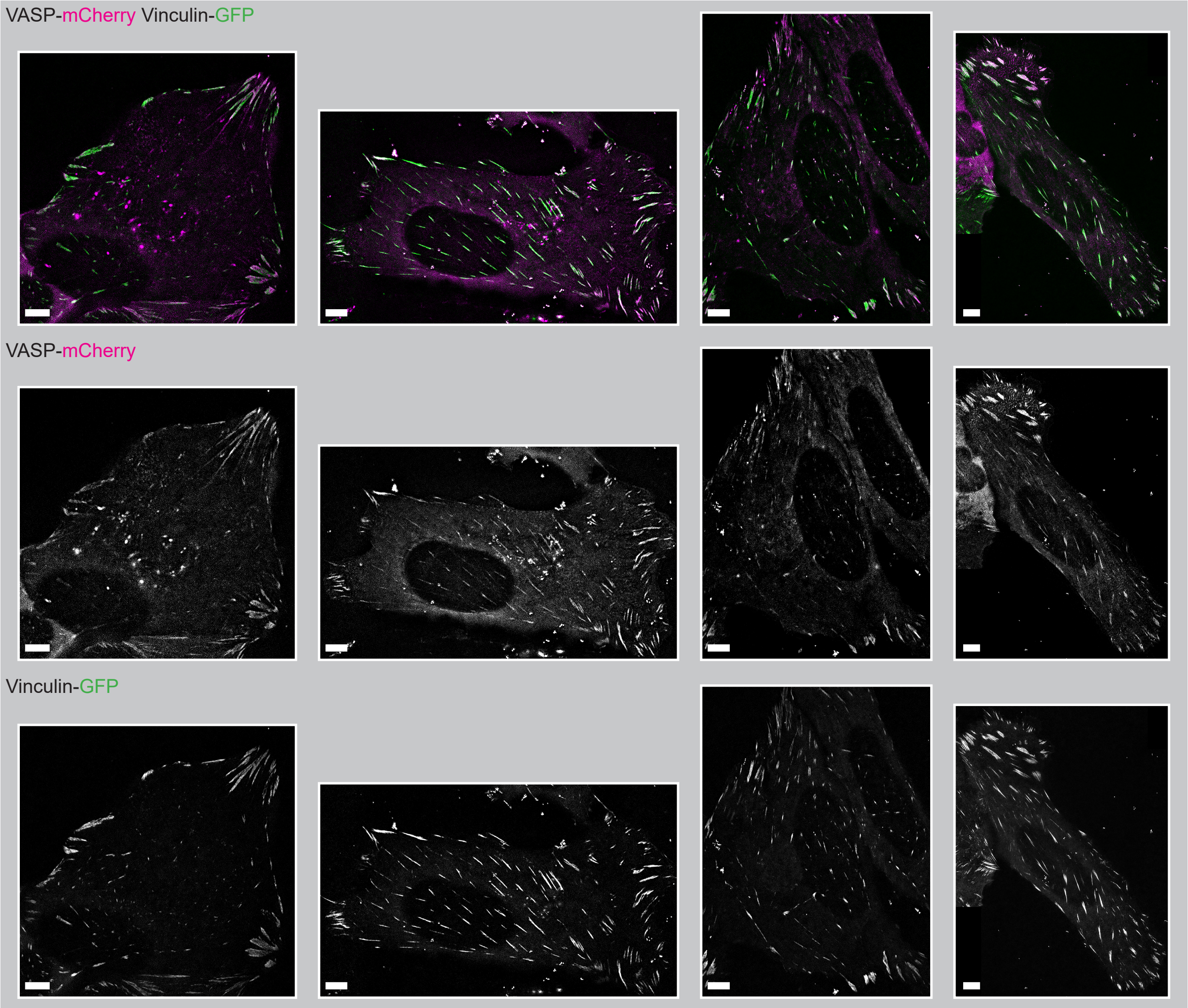

Fig. S6 Representative SIM images of U2OS cells coexpressing VASP-mCherry and Vinculin-GFP. Merged (top), red (middle) and green (bottom) channels, red channel is pseudocoloured magenta in merge images. Scale bar 5 µm. For visualisation purposes the contrast of the channels is enhanced (note that image analysis is performed on images with unaltered contrast settings).

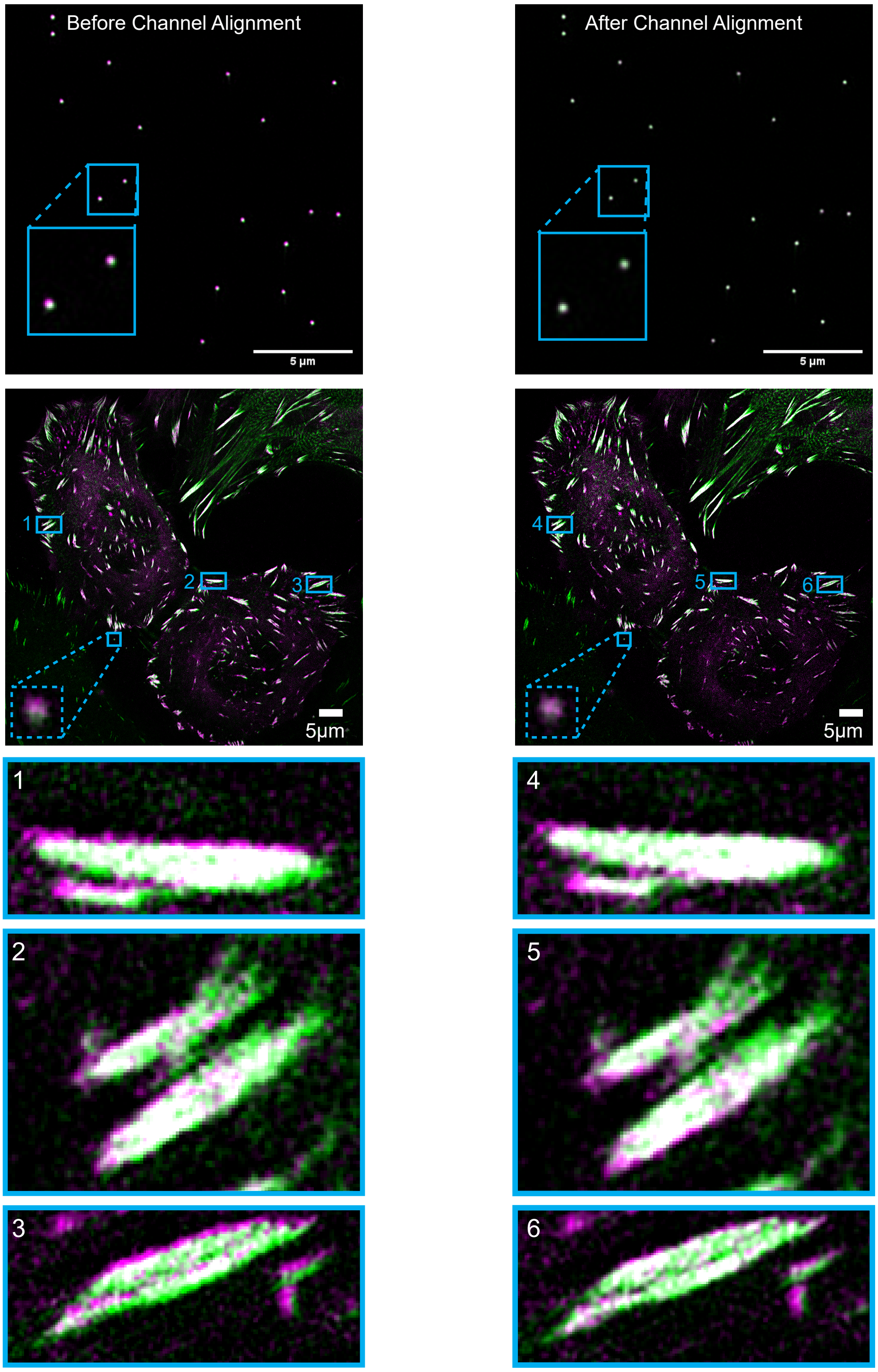

Fig. S7 Illustration of the channel alignment procedure applied to all SIM data prior to analysis. Merged SIM images of the red (pseudocoloured magenta) and green channels before (left) and after (right) channel alignment procedures. For visualisation contrast is enhanced (note that data analysis is performed on images with original contrast settings). Top panels show 100 nm multicolour TetraSpeck beads, as are used for chanel alignment. Panels below show U2OS cells coexpressing paxillin-mCherry and zyxin-GFP. Dashed box provides a magnification of the indicated area with a TetraSpeck bead used as an internal control. Numbered panels with blue outlines are magnifications of the corresponding numbered blue boxes in the images above.

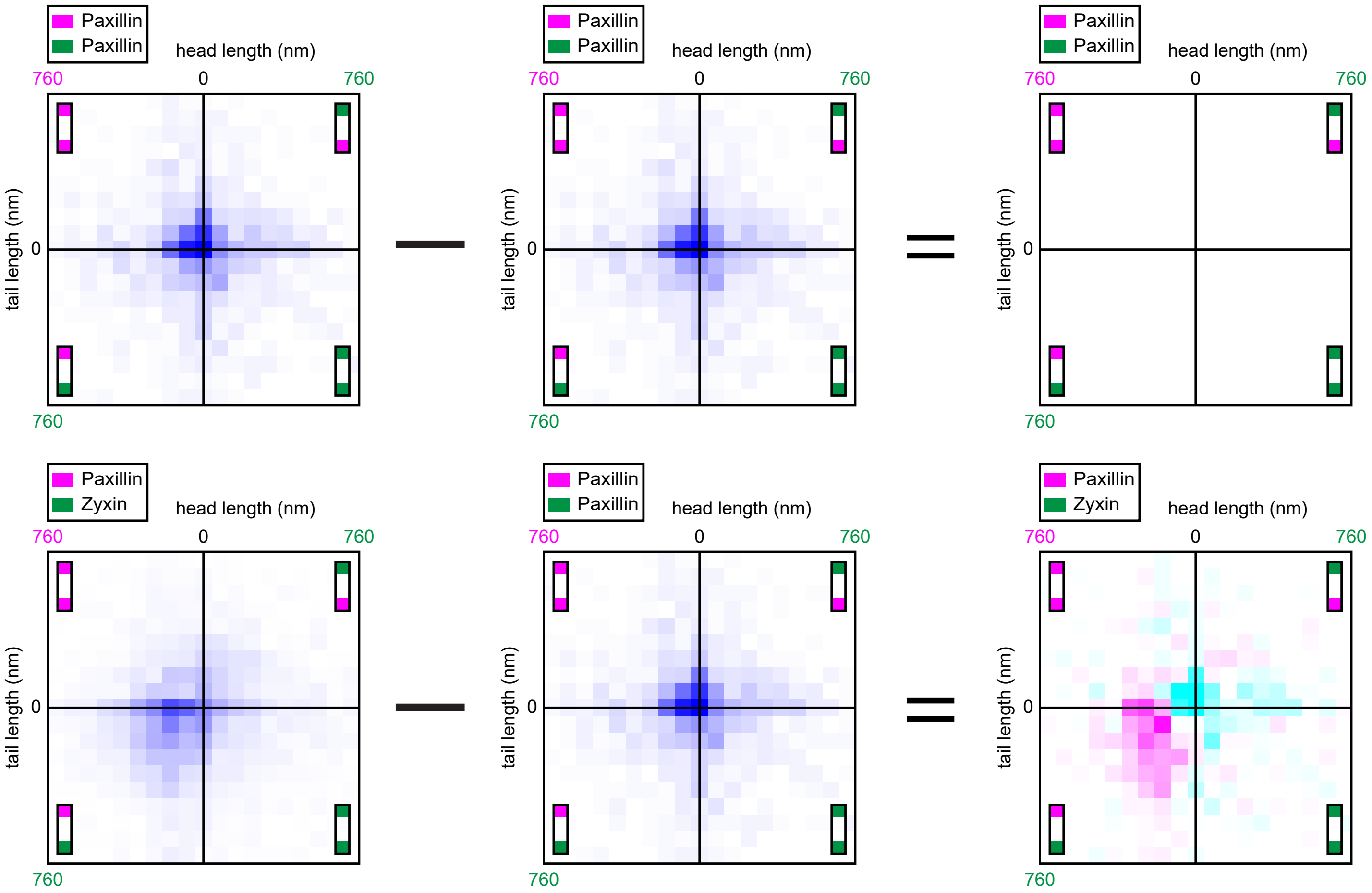

Fig. S8 Illustration of the method used to generate the 2D-histograms.
As a first step the separate head and tail histograms are combined into a single 2D-histogram which combines both head (horizontal axis) and tail (vertical axis) protrusion lengths for each individual FA in both the dataset of interest and the pax/pax reference set (blue histograms). Colour intensity represents the percentage of FAs within each bin, with a scale ranging from fully white at 0% to true blue at 50%. To generate the magenta and cyan 2D-histograms, the pax/pax reference 2D-histogram is substracted from the 2D-histogram of the dataset of interest. The magenta and cyan 2D-histograms show the difference between the relative distributions in the dataset and the pax/pax reference set (in percentage points). Cyan represents negative values, magenta positive values and white values below threshold, colour intensity represents magnitude of the difference (see Materials and Methods for threshold and scale).

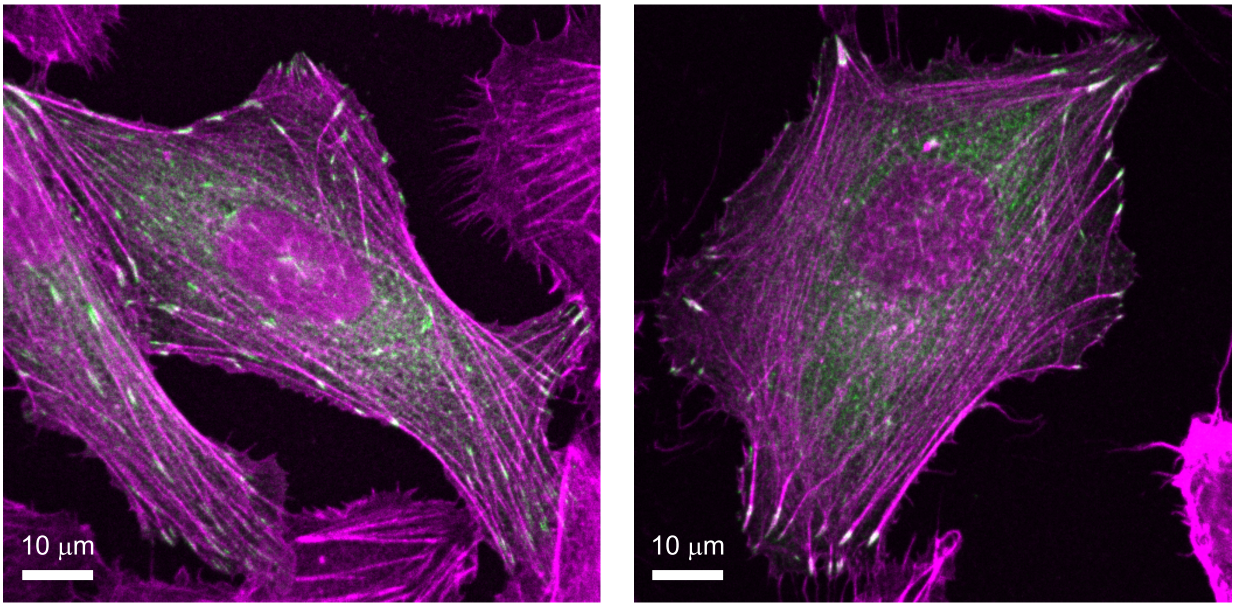

Fig. S9 F-actin stress fibres typically enter FAs at their tails.
Merge confocal images of U2OS cells stably expressing paxillin-GFP (green) stained with phalloiding-CF405 to highlight the F-actin (pseudocolour magenta).
